# The role of resilience in the relationship between role stress and psychological well-being during the COVID-19 pandemic: a cross-sectional study

**DOI:** 10.1186/s40359-023-01082-w

**Published:** 2023-02-14

**Authors:** Anita Padmanabhanunni, Tyrone B Pretorius, Natasha Khamisa

**Affiliations:** 1 Department of Psychology, University of the Western Cape, Bellville, South Africa; 2 School of Public Health, University of the Witwatersrand, Johannesburg, South Africa; 3 University of the Western Cape, Private Bag X17, 7535 Bellville, Republic of South Africa

**Keywords:** Indigenous model of stress-mental health, Resilience, Mediation, Role stress, Psychological well-being

## Abstract

**Background:**

Stress resistance resources, such as social support and resilience, have been found to be important in promoting psychological well-being during the COVID-19 pandemic. However, most prior research studies have conceptualized stress resistance resource variables as having a mediating or moderating role. Cooper (2018) proposed a model of the relationship between stress and health which posits that coping resources are always present and not only invoked in the face of adversity. Thus, we hypothesize that coping resources are causally antecedent to stressors and influence well-being indirectly via the stressor. We focused specifically on school teachers due to them being at the frontlines of service provision during the pandemic. Teaching was already identified as a highly stressful profession prior to COVID-19 and disease containment measures placed additional strain on teachers who had to adapt to emergency remote teaching.

**Aim:**

The current study tests this hypothesis by examining the indirect effects of resilience on indices of psychological health via role stress.

**Methods:**

Participants (*N* = 355) were teachers who completed the Connor-Davidson Resilience Scale-10, the Role Stress Scale, the Satisfaction with Life Scale, the State-Trait Anxiety Inventory-Trait Scale, and the Center for Epidemiological Studies Depression Scale. An electronic version of the questionnaires was distributed to teachers via Facebook and to officials from the Department of Education, who assisted with distribution of the electronic link to the survey. Participants were mostly women (76.6%) and mean number of years in the teaching profession was 15.7.

**Results:**

Structural equation modelling results demonstrated significant direct effects of resilience on life satisfaction, anxiety, and depression, which indicates that resilience is beneficial for psychological health even in the absence of stress. Resilience also had a significant indirect effect on indices of psychological well-being via role ambiguity but not role conflict.

**Conclusion:**

These findings have theoretical implications for the understanding of the role of resilience in promoting psychological health among educators. Practical implications include an empirical contribution to education policy and information that can inform interventions aimed to promote resilience among educators.

## Introduction

In South Africa, as in the rest of the world, the COVID-19 pandemic has severely impacted schooling, leading to severe consequences for teachers and pupils. When the pandemic first hit South Africa in March 2020, the government swiftly introduced a 5-level lockdown system, in which alert level 5 is the most restrictive and alert level 1 the least restrictive. Level 5 features drastic lockdown measures intended to contain the spread of the virus, including complete school closures [1]. Over the subsequent two years, the country rotated between the various lockdown levels depending on the spread of the virus and the health system’s readiness to deal with the pandemic. During this period, teaching and learning were limited as the government introduced various measures to protect teachers and learners, including a rotational learning system in which pupils attended school on alternate dates.

These disruptions to learning led to an unprecedentedly chaotic situation for teachers as their formerly structured work suddenly became unstructured, and the situation may have been exacerbated for many teachers due to lack of communication or miscommunication. In such a context, there is a significant likelihood of role stress for teachers. Role stress theory proposes that all individuals occupy roles that are associated with a range of expectations. When these expectations are conflicting or ambiguous, it leads to role conflict and role ambiguity [2, 3]. If the expectations associated with a role are incompatible, the result is role conflict, whereas expectations that are inconsistent, confusing, or unclear lead to role ambiguity [3]. Role conflict and role ambiguity have been associated with a range of adverse work-related behaviors (e.g., work engagement[4], job satisfaction and turnover intention [5], job performance [6]), psychological well-being [7–9], and burnout [4, 6, 10]. Mérida-López and Rey [8], in a pre-pandemic study, found that role conflict and role ambiguity were positively related to adverse mental health outcomes including depression, anxiety and stress among a sample of Spanish school teachers. Similarly, [9] reported that high role conflict and high role ambiguity along with low social support was associated with depressive symptoms among Japanese school teachers.

Different individuals who are exposed to the same levels of stress or adverse circumstances will not necessarily have the same reaction to these negative environmental conditions (e.g., negative psychological health outcomes or burnout). Differential vulnerability is the concept that personal and environmental coping resources ensure a differentiated response to adverse circumstances. Examples of the variables that make individuals differentially vulnerable to adverse conditions include social support [11, 12], willingness to use social support [13], locus of control [14, 15], problem-solving appraisal [16, 17], appraisals of safety [18], and career calling [6]. These variables are conceptualized to have either a direct, moderating, or mediating effect.

The direct effect hypothesis proposes that coping resources or protective factors have a direct relationship with psychological well-being that exists independently of the level of adversity experienced by an individual. The direct effect hypothesis is also referred to as the health-sustaining model [19]. In its simplest formulation, the health-sustaining model posits that the coping resource or protective factor does not only operate under adverse conditions; rather, having high levels of these resources is generally good for psychological health. Moderator variables interact with adverse circumstances to impact psychological health. A variable is said to operate as a moderator if the negative association between the adverse condition and psychological health decreases as the level of the coping resource increases. A mediator variable is a pathway through which the adverse condition impacts psychological health. In a mediated pathway, the adverse condition is causally antecedent to the mediator [20].

The construct of resilience has not been well-defined in the literature with some studies conceptualizing it as a “trait” which is stable and enduring while others view it as a “state” phenomenon which is dynamic and malleable. Recent definitions of resilience view it as a dynamic process through which the individual positively adapts to stressful events or adverse circumstances [21]. For the present study, we adopt Fergus and Zimmerman’s [22] conceptualization which separates resilience into personal assets (e.g., emotional regulation ability, tendency to use active coping strategies, self-esteem, locus of control, etc.) and resources which are external to the individual (e.g., social support networks). The process of resilience entails the individual utilizing both assets and resources to cope with stressors and achieve positive outcomes.

Resilience has been related to role conflict in several studies [23, 24]. One study found that resilience weakened the negative effect of role conflict on the performance of frontline service providers [25]. De Clercq [26] found that high resilience reduces role ambiguity. Employees with high resilience have high energy levels; in the absence of resilience, that energy would contribute to a stress response due to a lack of information about role descriptions. Researchers have also suggested that resilience positively contributes to life satisfaction [27], and some studies have indicated that resilience plays a mediating role in the relationship between career adaptability and life satisfaction [28]. Moreover, resilience has been found to be inversely associated with depression and anxiety among individuals affected by the COVID-19 pandemic [29]. Resilience may also be a protective factor for anxiety and depression due to resilient individuals’ ability to retain a positive attitude or outlook despite life-threatening events [30].

The direct, moderating, and mediating roles of protective factors have been the most common focus of studies on job stress; however, Cooper and colleagues [31, 32] proposed and found empirical support for an “indigenous” model of the relationship between stress and physical and psychological health, in which coping resources are causally antecedent to job stressors, which implies that coping resources determines how stress is experienced, which in turn positively impacts psychological well-being. Since the concept of an “indigenous” model might be slightly misleading we hereafter refer to it as the Cooper model. This model proposes that coping resources are always present and influence the ways in which stressors are perceived or managed. Moyle [33] refers to the Cooper model as the indirect effects hypothesis and argues that people bring certain personality dispositions to the work place that influence their experiences and interpretations of the work setting. In the Cooper model or indirect effects hypothesis, the mediated pathway is work-related stress rather than coping resources.

Protective factors, including resilience, have been found to positively affect psychological well-being [26]. Yildirim [34] found positive associations between resilience and life satisfaction, positive affect, affect balance, and flourishing. Social support has been associated with high resilience to stress [35], which may lead to improved psychological well-being. Other protective factors include locus of control [15] and sense of coherence [36], both of which have been found to be beneficial for psychological well-being.

The current study used path analysis to examine the indirect role of resilience in psychological well-being. Resilience is operationalized as a predictor variable that indirectly impacts psychological well-being via role stress as the mediated pathway (Cooper model). The indices of psychological well-being in the current study include life satisfaction, anxiety, and depression. The following direct effects hypotheses are examined:

H1: High levels of resilience will be associated with low levels of role conflict and role ambiguity.

H2: High levels of resilience will be associated with high levels of life satisfaction, low levels of anxiety and low levels of depression.

The negative effect of adverse factors on psychological well-being has been well documented in the literature [37], and several studies have demonstrated the mediating role of resilience, as reported in a systematic review by Li and Hasson [38]. For example, Zeng and colleagues [39] found that resilience mediated the relationship between mindset and psychological well-being in a sample of students. Another study found that resilience fully mediated the relationship between fear of happiness and life satisfaction [34]. Resilience has also been found to mediate the relationship between traumatic events and post-traumatic stress disorder [40]. However, it is important to note that in these studies, the adverse factor was presumed to be antecedent to resilience. Based on the Cooper model, we propose that resilience is not only invoked in the presence of adversity but is always present; therefore, it influences the way in which an adverse event or stressor is perceived and managed, which in turn impacts psychological well-being. We therefore propose the following hypotheses related to the Cooper model:

H3: High levels of resilience will be associated with low levels of role conflict, which in turn will be associated with low levels of anxiety, low levels of depression and high levels of life satisfaction.

H4: High levels of resilience will be associated with low levels of role ambiguity, which in turn will be associated with high levels of life satisfaction low levels of anxiety, low levels of depression and high levels of life satisfaction.

## Method

### Participants

School teachers were recruited to participate in the study. To be included in the study, they needed to have been active professionally during the pandemic. The study respondents (*N* = 355) were school teachers in South Africa. The majority of respondents worked at schools in the Western Cape Province (82.3%) and were women (76.6%). Their mean number of years in the teaching profession was 15.7 (*SD* = 11.8, range: 23–73) and the majority of respondents taught at primary schools (61.1%). The mean age of the sample was 41.9 years (*SD* = 12.4, range: 1–48). A fuller description of the sample is presented in Table 1.

**Table 1:** Description of sample

Variable	Category	N	%
Gender	Women	273	76.9
	Men	82	23.1
School type	Public	313	88.2
	Private	18	5.1
	Model C*	24	6.8
Area school located	Rural	169	47.6
	Urban	186	52.4
Area of residence	Rural	136	38.3
	Urban	219	61.7
Grades teach	Pre-primary	14	3.9
	Primary	217	61.1
	Secondary	122	34.4
	Learning support	2	0.6
Lost family due to COVID-19	Yes	128	36.1
	No	227	63.9
Lost colleagues due to COVID-19	Yes	96	27.0
	No	259	73.0
Age		Mean = 41.89	*SD* = 12.42
Years teaching		Mean = 15.70	*SD* = 11.75
Class size		Mean = 35.62	*SD* = 10.69

* refers to schools that under Apartheid were white-only schools

### Measures

Respondents completed the short form of the Connor-Davidson Resilience Scale (CD-RISC-10 [41]), the Satisfaction with Life Scale (SWLS [42]), the State-Trait Anxiety Inventory-Trait Scale (STAI-T [43]), the Center for Epidemiological Studies Depression Scale (CES-D [44]), the Role Stress Scale [3]), and a brief demographic survey. The original version of the CD-RISC consists of 25 items. However, the short 10-item form has been found to be a reliable and valid measure of resilience [45–47]. In these studies, reliability coefficients (Cronbach’s alpha) ranged from 0.83 to 0.88, which reflects satisfactory internal consistency. The association between resilience and similar constructs—such as ego resilience, self-efficacy, and psychological well-being—supports the validity of the CD-RISC-10. Responses to the CD-RISC-10 items are scored on a 5-point Likert scale with options ranging from *not true at all* (0) to *true nearly all of the time* (4). The CD-RISC-10 has been used in South Africa studies [48, 49] that have reported Cronbach’s alphas of 0.80 and 0.95, respectively. Pretorius and Padamanabhanunni [49] also found strong evidence for the unidimensionality and validity of the CD-RISC-10 using both classical test theory and item response theory.

The SWLS comprises 5 items that are used to measure the cognitive component of subjective well-being [50]. The items are scored using a 7-point Likert scale (1 = *strongly disagree*, 7 = *strongly agree*). The SWLS has been translated into many languages and used in various geographical contexts. Overall, sufficient evidence of the reliability, validity, and unidimensionality of the SWLS has been reported [51, 52]. In South Africa, Padmanabhanunni and Pretorius [53] reported satisfactory reliability estimates (*α* and *ω* = 0.89) for the SWLS among a sample of young adults.

The STAI-T is a measure of trait anxiety consisting of 20 items that are assessed on a 4-point Likert scale that ranges from *almost never* (1) to *almost always* (4). Evidence for the reliability and validity of the STAI-T has been well documented [54–56]. Padmanabhanunni and Pretorius [57] reported a satisfactory Cronbach’s alpha of 0.92 for the STAI-T among a sample of university students in South Africa.

The Role Stress Scale measures occupational stress and assesses two components of role stress, namely role conflict (RC) and role ambiguity (RA). The scale consists of 14 items, 8 of which measure RC and 6 of which measure RA. RC refers to the discordance between expectations of the role, whereas RA refers to uncertainty about the required actions to meet the expectations of the role [58]. The items of the Role Stress Scale are scored on a 6-point scale that ranges from *definitely not true of my job* (1) to *definitely true of my job* (6). Recent studies have reported satisfactory estimates of internal consistency for the Role Stress Scale [59, 60]; however, comprehensive assessments of the psychometric properties of the scale are outdated. Research studies from the 1990s have confirmed the reliability and validity of the Role Stress Scale [61–63].

### Procedure

The period of data collection for the current study was May–July 2021, which coincided with the period when the Delta variant was prevalent. An electronic version of the questionnaires was compiled and distributed to teachers via a post on a Facebook group for South African school teachers. The post consisted of a brief explanation of the aims of the study, invited participation and provided the electronic link to the survey. In addition, we presented the aims and objectives of the study to officials from the provincial Department of Education, who assisted with distribution of the electronic link.

### Ethics

The institutional review board of the University of the Western Cape provided ethical approval for the study (ethics reference number: HS21/3/8). Participation was voluntary. The survey was anonymous, and access to the survey was granted only after participants provided informed consent. Given the nature of the questionnaires and the possibility that study participation might evoke distress, respondents were provided with the contact details for psychological support services in case such distress was experienced.

### Data analysis

IBM SPSS Statistics for Windows (version 27) was used to obtain the correlations between study variables, as well as the reliabilities (Cronbach’s alpha and McDonald’s omega) and descriptive statistics. Structural equation modelling and bootstrapped confidence intervals (95%) were performed and calculated using IBM SPSS AMOS (version 27) to examine the direct and indirect effects of resilience on indices of psychological well-being.

## Results

The estimates of reliabilities, descriptive statistics, and intercorrelations are reported in Table 2. All the scales demonstrated satisfactory reliability (*α*: 0.82–0.95, *ω*: 0.83–0.95).

**Table 2:** Descriptive Statistics, Reliabilities, and Intercorrelations of Study Variables

	1	2	3	4	5	6
1. Resilience	—					
2. Role Conflict	0.01	—				
3. Role Ambiguity	−0.36^***^	0.04	—			
4. Life Satisfaction	0.45^***^	−0.09	−0.42^***^	—		
5. Anxiety	−0.53^***^	0.27^***^	0.34^***^	−0.52^***^	—	
6. Depression	−0.48^***^	0.23^***^	0.38^***^	−0.55^***^	0.74^***^	—
Mean	26.9	30.4	14.7	21.9	44.9	22.0
SD	8.0	8.2	5.7	7.3	10.3	12.2
Alpha	0.95	0.82	0.83	0.90	0.91	0.92
Omega	0.95	0.83	0.83	0.90	0.91	0.93

^***^p < 0.001

Role ambiguity was positively associated with anxiety (*r*_353_ = 0.34, *p* < 0.001, 95% CI [0.25, 0.43]) and depression (*r*_353_ = 0.38, *p* < 0.001, 95% CI [0.29, 0.47]) and negatively associated with life satisfaction (*r*_353_ = − 0.42, *p* < 0.001, 95% CI [0.29, 0.47]). The coefficients can be regarded as medium effect sizes. This finding indicates that high levels of role ambiguity were associated with high levels of anxiety and depression and low levels of life satisfaction. Role conflict was positively associated with anxiety (*r*_353_ = 0.27, *p* < 0.001, 95% CI [0.17, 0.36]) and depression (*r*_353_ = 0.23, *p* < 0.001, 95% CI [0.12, 0.32]) but not associated with life satisfaction. In all instances the coefficients can be regarded as medium effect sizes. These findings indicate that high levels of role conflict were associated with high levels of depression and anxiety. Resilience was positively associated with life satisfaction (*r*_353_ = 0.45, *p* < 0.001, 95% CI [0.37, 0.53]) and negatively associated with anxiety (*r*_353_ = − 0.53, *p* < 0.001, 95% CI [− 0.60, − 0.45]) and depression (*r*_353_ = − 0.48, *p* < 0.001, 95% CI [− 0.56, − 0.40]). With the exception of the correlation between resilience and anxiety, the coefficients can be regarded as medium effect sizes. In the case of the association between resilience and anxiety, the coefficient can be regarded as a large effect size. Thus, high levels of resilience were associated with high levels of life satisfaction and low levels of anxiety and depression.

**Fig. 1:**
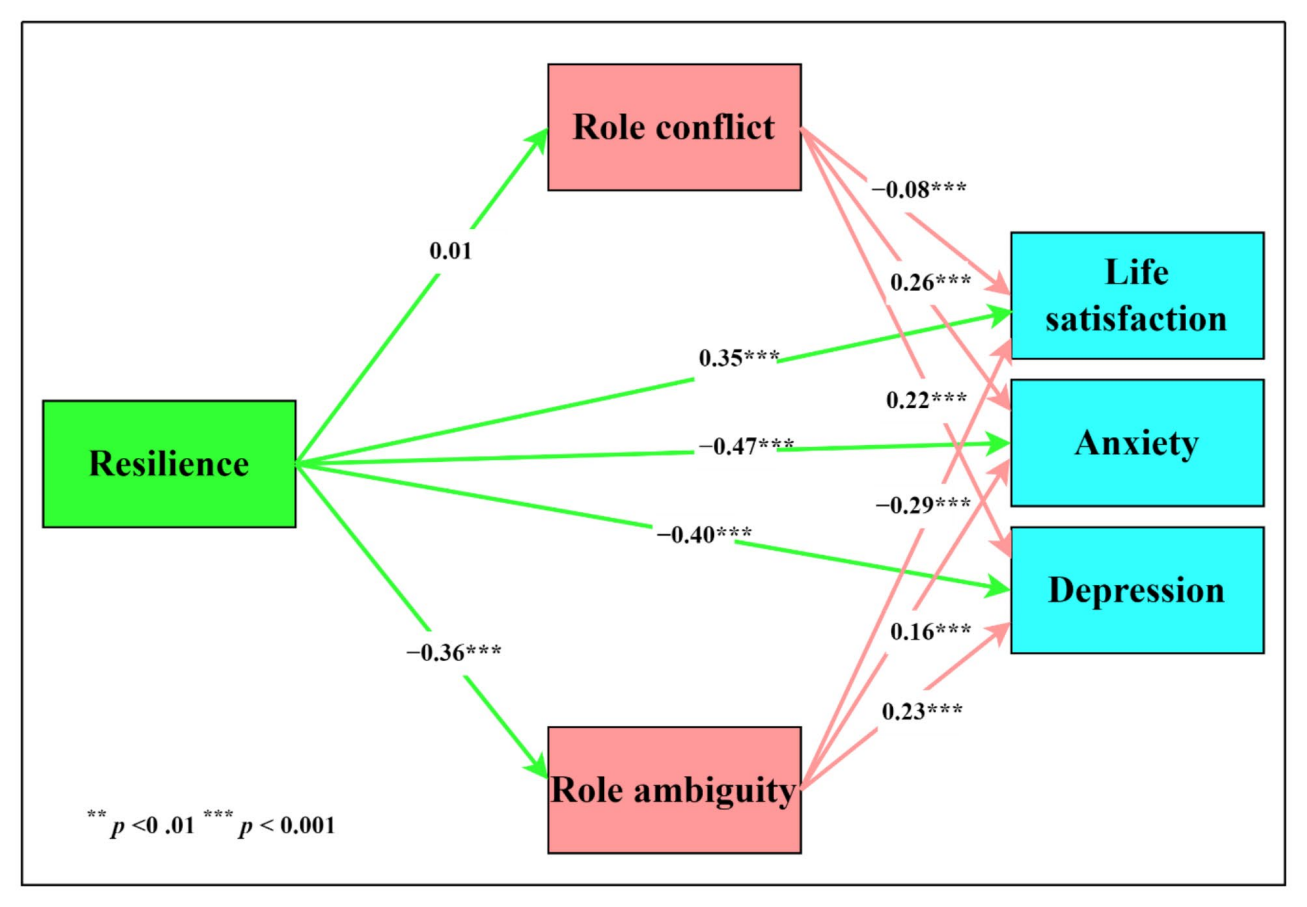
Path Analysis Model of the Direct and Indirect Effects of Resilience

The path analytical model that was used to examine the direct and indirect effects of resilience on indices of psychological well-being is presented in Fig. 1. In this model, resilience is conceptualized as the independent variable and indices of psychological well being as the dependent variable, while role stress is conceptualized as the pathway through which resilience impacts the indices of well-being.

The indices associated with the direct and indirect effects resulting from the model in Fig. 1 are reported in Table 3. These indices reflect that all the direct effects of resilience were significant except the relationship between resilience and role conflict. Resilience was positively associated with life satisfaction (*β* = 0.35, *p* < 0.001, 95% CI [0.27, 0.43]) and negatively associated with role ambiguity (*β* = −0.36, *p* < 0.001, 95% CI [− 0.45, − 0.28]), anxiety (*β* = −0.47, *p* < 0.001, 95% CI [− 0.55, − 0.40]), and depression (*β* = −0.40, *p* < 0.001, 95% CI [− 0.48, − 0.32]). All of the regression coefficients in respect of the direct effects can be regarded as medium effects. These findings partially support Hypotheses 1–2 regarding the direct effects of resilience, but they do not support the part of the hypotheses related to the association between resilience and role conflict.

**Table 3:** Direct and Indirect Effects of Resilience on Role Stress and Psychological Well-being

Effect	Beta	SE	*β*	95% CI	*p*
Direct Effects					
Resilience → RC^H1^	0.01	0.06	0.01	[− 0.07, 0.10]	0.86
Resilience → RA^H1^	−0.26	0.04	−0.36	[− 0.45, − 0.28]	0.00
Resilience → Satisfaction^H2^	0.32	0.04	0.35	[0.27, 0.42]	0.00
Resilience → Anxiety^H2^	−0.61	0.06	−0.47	[− 0.55, − 0.40]	0.00
Resilience → Depression^H2^	−0.61	0.07	−0.40	[− 0.48, − 0.32]	0.00
RC → Satisfaction	−0.07	0.04	−0.08	[− 0.16, − 0.01]	0.06
RC → Anxiety	0.33	0.05	0.26	[0.19, 0.33]	0.00
RC → Depression	0.33	0.07	0.22	[0.15, 0.29]	0.00
RA → Satisfaction	−0.38	0.06	−0.29	[− 0.37, − 0.20]	0.00
RA → Anxiety	0.30	0.08	0.16	[0.08, 0.24]	0.00
RA → Depression	0.50	0.10	0.23	[0.16, 0.31]	0.00
Indirect Effects					
Resilience → RC → Satisfaction^H3^	−0.01	0.01	−0.00	[− 0.01, 0.01]	0.76
Resilience → RC → Anxiety^H3^	0.00	0.02	0.00	[− 0.03, 0.04]	0.85
Resilience → RC → Depression^H3^	0.00	0.02	0.00	[− 0.03, 0.04]	0.85
Resilience → RA → Satisfaction^H4^	0.10	0.02	0.11	[0.06, 0.14]	0.00
Resilience → RA → Anxiety^H4^	−0.08	0.02	−0.06	[− 0.12, − 0.04]	0.00
Resilience → RA → Depression^H4^	−0.13	0.03	−0.08	[− 0.19, − 0.08]	0.00

*Note.* Superscript notation refers to hypothesis numbering. Satisfaction = Life satisfaction, RC = Role conflict, RA = Role ambiguity

Regarding the indirect effects of resilience, Table 3 reflects that the role ambiguity paths were significant, whereas the role conflict paths were not significant. Resilience indirectly impacted life satisfaction (*β* = 0.11, *p* = 0.001, 95% CI [0.06, 0.14]), anxiety (*β* = −0.06, *p* = 0.003, 95% CI [− 0.12, − 0.04]), and depression (*β* = −0.08, *p* = 0.001, 95% CI [− 0.19, − 0.08]). These findings support Hypothesis 4.

## Discussion

The aim of this study was to explore the indirect effects of resilience on indices of psychological well-being via role stress. The majority of research on resilience during the pandemic has investigated its mediating role in the association between stress and burnout, stress and quality of life and social isolation and wellbeing [64–66]. Furthermore, these studies on resilience have largely focused on frontline medical care workers with few studies investigating resilience among other population groups differentially impacted by the pandemic. This study aims to extend research on resilience through its focus on school teachers and its role in the relationship between role stress and indices of psychological well-being [38, 67]. Role ambiguity was negatively associated with life satisfaction and positively associated with anxiety and depression. Role conflict was positively associated with anxiety and depression. The structural equation modelling results demonstrated significant direct effects of resilience on psychological well-being, which highlights the health-sustaining role of resilience. These findings indicate that resilience is beneficial even in the absence of adverse circumstances or negative events.

Consistent with the Cooper model of the stress and health relationship, resilience indirectly impacted psychological well-being via role ambiguity. Thus, resilience was found to be antecedent to role ambiguity. As explained by Cooper and Baglioni [32], this finding indicates that resilience is not only invoked in times of adversity but is always present. An individual’s level of resilience determines whether they experience their work environment as ambiguous, which in turn is associated with psychological well-being.

A further finding was that resilience did not impact psychological well-being via role conflict. It is probable that teachers in the study were able to rationalize that the COVID-19 pandemic placed additional demands on all frontline workers and this meant greater responsibilities. This finding is similar to that of a study of a U.S. disaster response workforce, in which role ambiguity was found to be a stronger predictor of psychological well-being (specifically burnout) than role conflict [68]. New and exacerbated challenges brought about by the COVID-19 pandemic and the unclear role expectations established in responding to the pandemic might explain the significance of role ambiguity in predicting psychological well-being. Alyahya and colleagues [69] have argued that continuous changes in COVID-19 policies have likely created role ambiguity, which makes it difficult for individuals to adapt to rapid changes that affect their roles. Worlu and colleagues suggest that effectively classifying roles may assist in dealing with role ambiguity [70]. In this regard, they suggest that roles where the confines and jurisdiction are clearly delineated, will assist in reducing role ambiguity. For role conflict, they suggest that role scheduling, whereby expectations about one’s role do not clash, has been found to reduce role conflict and relieve the pressure that results from role conflict [70]. However, role conflict has also been found to be positively and directly associated with creativity by stimulating receptive and divergent ways of thinking [71]. Several studies have confirmed that role conflict encourages flexible thought processes and allows for the generation of new ideas [72, 73]. This is achieved through high levels of mindfulness, which is beneficial for making cognitive adjustments at work. A mindful employee is motivated to use energy to obtain work-related knowledge and skills, which are then used to invoke creative responses to conflicting role demands [71].

De Clercq [26] has suggested that the uncertainty stemming from lack of information about job responsibilities is minimized when resources related to resilience, task interdependence, and emotion sharing are present. Resilience enables individuals to cope with ambiguous work situations because they can view such situations as learning opportunities, and task interdependence demonstrates the interconnectedness between tasks performed at work, which allows employees to learn from each other. Emotion sharing describes the extent to which employees share their emotional experiences with each other, which promotes healthy functioning [26]. These personal, task-based, and relational resources invoke positive energy that enable individuals to engage in creative behaviors despite role ambiguity [74].

### Limitations

The study has certain limitations. First, the use of an electronic survey may have led to response bias in that respondents who were more familiar with information communication technology may have been more inclined to respond than others. Second, the use of a cross-sectional design limits the conclusions that can be made about causal relationships. A longitudinal analysis is necessary to examine the causal processes that underlie the relationship between resilience, role stress, and indices of psychological distress. Third, the sample was mostly comprised of female participants from one geographical location, and this lack of heterogeneity may limit the generalizability of the results. Finally, the data was collected during the COVID-19 outbreak and it is probable that the results do not reflect the interactions between these variables in non-pandemic conditions.

### Conclusion

The current study examined the indirect effects of resilience on indices of psychological health via role stress among a sample of school teachers. Even when work-related stressors are absent resilience was found to be a protective factor that had a significant indirect effect on indices of psychological well-being via role ambiguity but not role conflict. Accordingly, policy makers and management teams should be proactive in reducing sources of role ambiguity for teachers. Management teams could foster resilience through knowledge sharing, provision of written documentation detailing roles, and promotion of awareness of the inter-relationships between job tasks.

## Data Availability

All data generated or analyzed during this study are included in this article.
